# Griefing reaction and social networks

**DOI:** 10.1192/j.eurpsy.2022.1478

**Published:** 2022-09-01

**Authors:** M.D.C. Molina Liétor, M. Martín, I. Cuevas Iñiguez, I. Romero Gerechter, A. Sanz Giancola

**Affiliations:** 1 Hospital Universitario Príncipe de Asturias, Psiquiatría, Alcalá de Henares, Spain; 2 Hospital Universitario Príncipe de Asturias, Psychiatry, Alcalá de Henares, Spain

**Keywords:** Instagram, Griefing reaction, Facebook

## Abstract

**Introduction:**

Grief is a normal and not necessarily pathological psychological process that occurs after the loss of a family member or loved one with its psycho-affective consequences, external manifestations and rituals. Although mourning processes can be associated with losses of different types (employment, housing, baseline situation, housing), we will refer to mourning for the loss of a loved one. For some people, social networks facilitate the expression of feelings and experiences of grief, connecting with the emotional support of other friends and loved ones. However, the presence of accounts belonging to these deceased persons, the persistence of photos and memories that periodically appear on the screen without the person being able to choose them, can make it difficult to process the mourning.

**Objectives:**

The aim of this paper is to consider the beneficial and detrimental factors of social media during a grieving reaction after the loss of a loved one.

**Methods:**

For the preparation of this work, a bibliographic review on the subject has been carried out. Likewise, the clinical information provided by patients during our evaluations has provided critical views on what has been published in this regard.

**Results:**

Support through social networks can help to feel more affectionate, but there are other harmful factors that must be taken into account: permanence of photos, appearance of memories and reminder of the deceased person’s birthday.

**Conclusions:**

Social networks can have favorable but also detrimental factors in the elaboration of a grief and should be considered in the psychiatric exploration and intervention.

**Disclosure:**

No significant relationships.

